# Systematic Review of the Combined Treatment of Endo, Ortho and Prosthesis in Maxillary Central Fracture Based on Surgical Points: Combined Treatment of Endo, Ortho and Prosthesis in Maxillary Central Fracture

**DOI:** 10.31661/gmj.v13iSP1.3653

**Published:** 2024-12-08

**Authors:** Sajedeh Safari, Anahita Dehghani Soltani, Mohammad Amin Bafandeh, Amir Abolhasani Zratcar, Nadieh Qaderi

**Affiliations:** ^1^ Prosthodontic Department, Faculty of Dentistry, Tehran Medical Sciences, Islamic Azad University, Tehran, Iran; ^2^ Department of Orthodontics, School of Dentistry, Shahid Beheshti University of Medical Sciences, Tehran, Iran; ^3^ Department of Prosthodontics, Faculty of Dentistry, Shahed University, Tehran, Iran; ^4^ Department of Endodontics, Faculty of Dentistry, Shahed University, Tehran, Iran; ^5^ School of Dentistry, Tabriz University of Medical Sciences, Tabriz, Iran

**Keywords:** Combined Treatment of Endo, Ortho and Prosthesis, Maxillary Central Fracture, Surgical Tips, Dentist, Tooth Destruction

## Abstract

**Background:**

The present study systematically investigated the combined treatment of endo, ortho and prosthetics in maxillary central fractured teeth based on surgical points.

**Materials and Methods:**

In the current study, the issue was investigated by reviewing 39 articles and considering key words such as: “Combined endo, ortho and prosthetic treatment”, “Central fractured tooth”, “Upper jaw”, “Surgical tips”. The treatment of root-treated teeth whose crown structure has suffered severe destruction has always been considered.

**Conclusion:**

Composite blinds along with cemented tooth-colored dowels are commonly used to restore root-treated teeth, and many studies have been done on the mechanical properties and to some extent micro leakage of these restorations. One of the main causes of failure in restorative treatments for endodontic teeth with extensive destruction is the reduction of tooth fracture resistance and restoration.

**Results:**

The results of the recent study showed that the angles that in single units will cause the access hole to be placed in the buccal and in bridges will cause the prosthesis to be locked in the connections.

## Introduction

Dental surgery includes any type of surgery that is performed on the jaw bones and teeth. Most of the time [1], when the dentist uses the term "Dental surgery", he faces a lot of panic and fear from his patients. The reason is that most patients consider this surgery to be a very painful and expensive process [2]. This surgery includes procedures that help treat many dental and jaw problems [3]. In most cases, this surgery is considered an outpatient procedure and patients can resume their normal activities within a few days [4].

Endodontics is a dental treatment during which a dentist specializing in denervation removes the infected root by creating access to the tooth pulp space and cleans and disinfects the inside of the tooth canal [5]. After that, fill the space inside the canal with special materials so that the interior space of the canal is protected from the penetration of bacteria. Ortho veneer is a combination of quick and short-term orthodontic treatment (3 to 6 months) and dental composite.

Having a beautiful smile can inspire us with a sense of self-confidence and special charm [6]. Today, many people in all age groups are looking to improve their smile design. The process of improving the smile design and creating a Hollywood smile is done by a cosmetic dentist through one or more cosmetic methods [7], including dental laminate, dental composite, orthodontics, etc. Ortho veneer is an innovative combination of quick and short-term orthodontic treatment with composite veneer [8], creating a unique and standard smile for patients. Our goal is to perform the best treatments with the best techniques, we must be careful about dental composite or dental laminate treatments [9], we cannot smooth every dental disorder (crading) with dental composite or with dental laminate, the standard is that only mild disorders dental cases are composite teeth or dental laminates, unfortunately [10], we see that severe disorders are also treated with dental laminates or dental composites without orthodontics [11], which causes irreparable damage to the teeth and gum tissue in the future [12].

To restore such teeth, patients and dentists have been looking for a method that has more durability and survival and is exempt from exorbitant costs and complicated procedures. It should be mentioned that the standard treatment for moderate to severe dental disorders is ortho veneer treatment [13].

Ortho veneer is done in such a way that first a short-term and fast orthodontics is performed for 3 to 6 months according to the severity of the dental disorder and the condition of the gums and jaw bone of the patient, and when the teeth are straight and the arch of the jaw is corrected in order to be fixed dental composite treatment is also performed and a wonderful result is obtained [14].

## Search Strategy and Selection of Articles

**Figure-1:**
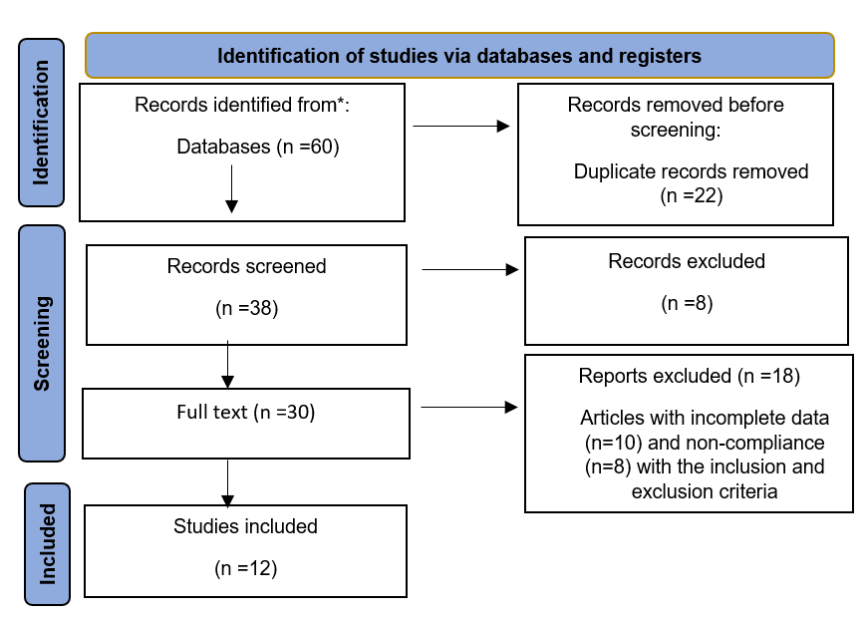
Figure 1. Flow PRISMA 2020 of included subjects

Search in Scopus, Google scholar, PubMed databases and by searching with keywords such as "Nursing Services", "Combined Treatment of Endo", "Ortho and Prosthesis", "Maxillary Central Fracture" and "Surgical Points" to obtain articles related to the selected keywords. Case report articles, editorials, and articles that were not published or only an introduction of them were available, as well as summaries of congresses and meetings that were in languages other than English, were ignored (Figure-1).

## Investigating methods of repairing broken teeth

It is better not to pull a broken tooth; tooth extraction is the last resort when none of the treatment methods work. If the crack or fracture of the tooth figis superficial and the necessary care is not taken, the root of the tooth may be damaged, and when the root of the tooth is damaged, the treatment procedures will be longer and the treatment costs will be higher [15]. There are different methods to repair a broken tooth, usually the dentist can use the best solution according to the type of fracture and the amount of damage [16]. If the necessary measures for recovery are done as soon as possible, the treatment will be associated with less pain and less costs [17].

## Bonding (composite) and coating for tooth fracture repair

Bonding is one of the suitable methods for repairing tooth fracture. This method can be used when a fracture occurs in the front teeth of the mouth [3]. The materials used to repair a broken front tooth can be different. Dentists prefer to use more types of composites. The use of this method, in addition to the beauty of the teeth, also provides the necessary resistance for chewing [18]. Covering is another method used to treat broken teeth. When a tooth is decayed or broken for any reason, it can be repaired using a veneer [19].

## Factors affecting the duration of orthodontics

In the following, we will mention the most important factors that affect the duration of orthodontics:

### Experience and skill of an orthodontic specialist

The first factor that we can introduce in this field is related to your dentist. The process of placing orthodontic brackets on your teeth, monthly check-ups can be one of the things that require a lot of time [2]. This is despite the fact that if you ask an expert and skilled orthodontist to take over your orthodontic treatment process, you will realize for yourself how much his experience and skills are effective in the speed of the treatment process [13]. Therefore, we can say that if you are one of the people who are concerned about the duration of orthodontics today and you intend to spend the minimum possible time for this issue in any situation, it is better to know that choosing a doctor can have a great impact on this matter.

### Type and severity of dental abnormalities

Abnormality severity is another important influencing factor during treatment. Usually, the length of treatment for patients with severe abnormalities is longer than for other patients. The more jaw abnormalities or jaw relations, the more time required for treatment [19]. If the misalignment of the teeth is mild to moderate, orthodontic treatment usually takes about a year [20].

### The speed of teeth movement

The speed of teeth movement is different for each person. Even in adulthood, many people’s teeth quickly respond to orthodontic brackets and shift [8]. Of course, there are some devices that stimulate the teeth to move more easily with high frequency vibration and bone regeneration around the root. In fact, they can shorten the duration of orthodontic treatment by 50%. The same devices are also used in new orthodontic methods in a short time [19].

### Orthodontic type

Today, orthodontic applicants have many options to choose from: Traditional metal braces, ceramic braces, and transparent braces are all types of orthodontics. Metal braces are made of solid color metal, which keeps this system very strong [21]. On the other hand, ceramic braces and clear aligners look more beautiful than metal braces and most adults prefer them [6]. The type of orthodontics is also one of the factors affecting the duration of orthodontic treatment [22].

### Taking some medications

With the excessive use of some drugs such as ibuprofen, aspirin or acetaminophen, the movement of the teeth slows down, and as a result, the duration of orthodontic treatment increases. Also, some other drugs such as thyroid drugs and vitamin D can make teeth move faster and reduce the duration of orthodontic treatment. Therefore, before you start your orthodontic treatment, provide your dentist with sufficient information about the specific medications you are taking [8].

### Age of the patient

Another factor that is very influential in the duration of orthodontics is the age of the patient. The younger the patient is, the sooner the treatment results. Also, the older the person, the longer orthodontics will take. Children’s jaws and teeth are growing [23]. Therefore, it does not take much time to straighten their teeth. The duration of teeth straightening in children’s orthodontics is less than in adults’ orthodontics [10].

### The intensity of the pressure applied by the brackets

The amount of pressure that orthodontic brackets apply to the teeth also affects the duration of orthodontic treatment. If the teeth have little crowding and crowding, orthodontic brackets and braces can improve the condition of the teeth in less than a year. But if it is the opposite of this story, it takes longer time to replace the teeth. Also, the arrangement of the teeth in acute cases has a great effect on prolonging the treatment process [1]. (Table-1).

**Figure:**
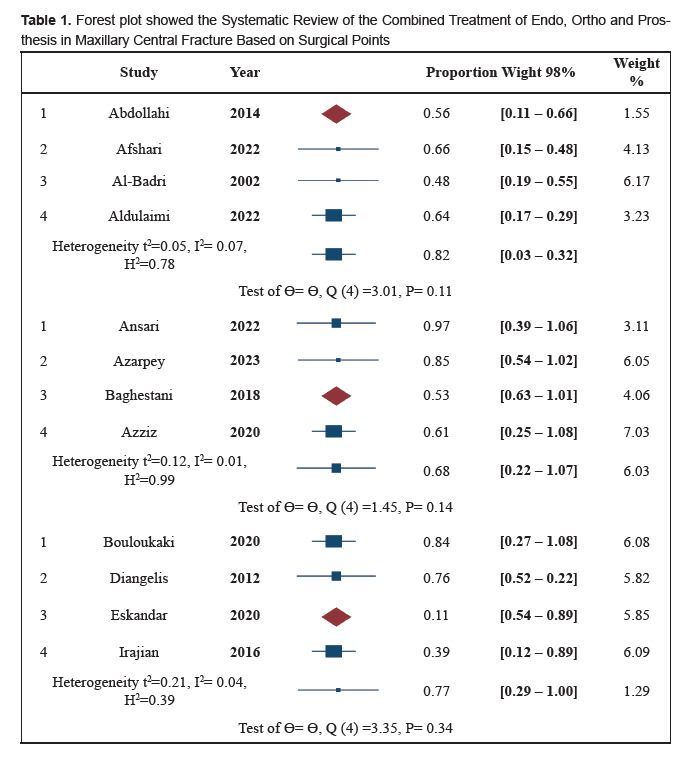


## Discussion

This disease is initially caused by a small inflammation in the pulp and even the tissues around the root, and is usually not accompanied by pain, but with its progress, irreversible damage to the pulp (usually accompanied by pain) and subsequently pulp necrosis and periapical diseases are created. Also, in cases where the deep caries of the crown has progressed to the dental pulp, with the diagnosis of the treating dentist [24], CEM cement can be used as an alternative to endo treatment. By using this method, the living tooth is saved [25], and as a result, the chance of the tooth lasting throughout life increases.

The first condition for the possibility of treatment with cement poison is that the dental pulp is alive and healthy [26];

In cases where the pulp of the tooth is dead due to trauma or progress of decay and infection inside the canal, it is not possible to treat the living pulp [27];

Also, in cases where a significant amount of tooth tissue has been lost due to decay and it is necessary to place a post and cover or a pin inside the canal, root canal treatment is mandatory [28];

CEM cement, which is made from a combination of a powder and a liquid, is a hydrophilic cement that after therapeutic use, its proper properties are intensified in the presence of water or humidity. This cement is synthesized from various calcium compounds such as calcium oxide, calcium phosphate, calcium carbonate, calcium silicate and calcium aluminate and it shows the following properties [29].

### Antimicrobial property of CEM cement with calcium

In terms of antimicrobial properties [30], CEM cement has been compared with hydroxide as one of the best disinfectants inside infected canals, as well as with MTA as one of the best materials used in specialized dental treatments, as well as with Portland cement.

### Flooding

The amount of micro leakage of CEM and its comparison with IRM and three types of American, Brazilian and Iranian MTA have been investigated as root end filling materials in various environments. The research results have shown that the amount of flooding caused by these materials is of course [31], the ability to cause flooding by cement poison and MTA was significantly higher than IRM. In another study, tooth root canals were filled using single cone gutta-percha cement, although the apical flood was similar to the MTA group, but the coronal flood was significantly better than the MTA group [32].

### Convenient clinical application

In a study that was conducted on different physical properties of cement poison and its comparison with MTA, it was shown that the working time and dimensional changes of cement poison and MTA were similar to each other. The setting time in cement resin was shorter than MTA, the amount of flow in cement resin was higher than MTA [33], and also the film thickness obtained in the case of cement resin was less than MTA, which are all very important advantages for the clinical use of a dental material.

## Biocompatibility and induction of construction of the ivory bridge

### Direct pulp coating

The results obtained from the application of cement poison as a material for directly covering the dental pulp and comparing it with calcium hydroxide and MTA indicate the formation of the dentin wall at a faster rate and with better structural integrity in the case of CEM and MTA, but in the case of calcium hydroxide of this wall is not formed completely [34].

### Pulpotomy

In a study that was conducted on dog premolar teeth, pulpotomy treatment was performed using cement poison, the samples were examined for the presence of quality, inflammation and thickness of the calcification barrier, pulp condition and morphology of odontoblasts. The results obtained in the cement poison group were significantly better than CH [35], but not significantly different from MTA. A rare report about maxillary central tooth pulpotomy with open apex exposed due to trauma after a period of one month in addition to the formation of Dentin Bridge under CEM material showed the results of successful apex genesis along with the construction of Dentin Bridge under cementum cement [36].

Also, in a study, twelve adult permanent molars suffering from irreversible pulp inflammation were treated with pulpotomy using cement poison, and for the first time to treat this disease, completely successful results were obtained after about 16 months with this technique and they showed a new material. In this study, it was shown that the pulp tissue of the human third molar tooth, by forming a calcified barrier under the cement, enclosed itself again in the internal environment of the tooth, and in other words, tissue regeneration has been achieved. This treatment method is the first time to propose living pulp treatment as a simple, cheap, and yet successful alternative treatment for irreversible pulp inflammation instead of the usual root canal therapy and has demonstrated its remarkable success [37]. In this way, most teeth can be treated using this new treatment method. However, this treatment is not recommended for teeth that require the use of their root canal space to place a pin inside the canal or a post for the final restoration of the crown.

### Production of hydroxyapatite

In addition to the mentioned characteristics, cement can produce hydroxyapatite crystals on its surface in normal saline environment. The crystal structure of the formed hydroxyapatite is similar to standard hydroxyapatite crystals. This property is not present in MTA material. Therefore, it can be concluded that the CEM dental material, unlike the MTA material, contains the chemical factors required for the formation of hydroxyapatite crystals. In addition [38], cement poison in the same environment as interstitial fluid (PBS) by producing more amounts of hydroxyapatite creates better conditions for increasing its flood as a filling material at the end of the root canal.

### How to use CEM cement

Before starting the treatment by direct observation or performing vital tests, it is necessary to make sure that the dental pulp is alive. Of course, the survival of the dental pulp should be removed in case of caries, infected enamel and dentin using a high-speed burr and frequent washing. It is better to use a tungsten carbide or coarse steel burr with a low speed and using a lot of water washing flow near the dental pulp [39]. It should be noted that isolating the treated tooth from the time of pulp exposure is necessary because with the above two operations, the aim is to remove pathogenic agents from the tooth cavity, and at the same time, we do not want contamination to occur again. Before exposing the pulp and before entering the pulp chamber, the dentist must make sure that firstly he has removed all the caries and secondly the shape of the cavity is complete for its final restoration. In the treatment of living pulp, the dentist must be very careful when removing dental caries so that no further damage is caused to the pulp. After removing the caries and cleaning the tooth crown, if the tooth is able to accept the treatment, the dentist should cover the pulp with a biomaterial such as calcium-enriched cement (CEM) and then repair the tooth crown [10].

CEM cement dental material is a suitable biological material for all types of living pulp treatments and it works in such a way that it can act as a non-porous coating after the treatment of pulp infection and inflammation by filling all the voids in the area. It creates a suitable covering barrier for the pulp that contains the nerves of the tooth root, and in this way, improves the toothache, while in this condition, the tooth pulp also survives and does not come out [39].

## Conclusion

In a recent study by the University of Minnesota, 196 single-tooth implants were compared with 196 root canal treatments. In this study, root-canalized teeth with veneers and single-tooth implants had the same failure rate of 1.6%. While the implant group had a longer mean and median in terms of performance, as well as the highest incidence of post-implantation complications, such as prosthetic problems that required additional follow-up treatments. Another point in the above review was the person responsible for root canal treatment or implant placement, which could be different from faculty members, residents, or general dentistry students to perform root canal treatment, to faculty members, oral and maxillofacial surgery residents, or perio for implant placement. Therefore, it is necessary to consider the necessary skill when deciding between implant placement or root canal treatment. Today, many dentists have joined the ranks of pure implant fans. But the purpose of this article is to understand the merits of a good root canal treatment. It is acceptable that there are cases where an implant is undoubtedly the best treatment. But have we reached the point where we no longer think about clinical crown lengthening as a treatment plan? Also, tooth extrusion is a very desirable treatment for some single-rooted teeth in many cases. Because it requires minimal surgery, faster time for dental function, less risk of treatment and a more appropriate cost for the patient. Endo is related to prosthesis. If the root canal treatment is done on the tooth and it is assumed that the post cannot be contracted and repaired, it can practically be said that nothing has been done. The purpose of root canal treatment is not to clean, shape and fill the root canal system. The main idea is to perform root canal treatment in such a way that the tooth can be repaired later and the patient’s occlusion can be restored. This is the same problem as it is seen that the majority of root canal treatment failures occur in the coronal one-third to the apical one-third region of the root. Implants have been placed. The main cause of this failure is excessive removal of dentin and widening of the coronal third of the root canal and weakening of the tooth, the main cause of these failures. Excessive coronal shaping of the root results in more complications during post-core placement (fractures). The tooth becomes susceptible to coronal leakage, recurrence of caries and endangering the whole structure of the coated tooth.

## Conflict of Interest

None.
